# A novel method for biosynthesis of different polymorphs of TiO_2_ nanoparticles as a protector for *Bacillus thuringiensis* from Ultra Violet

**DOI:** 10.1038/s41598-019-57407-6

**Published:** 2020-01-16

**Authors:** Elham Jalali, Shahab Maghsoudi, Ebrahim Noroozian

**Affiliations:** 1 Department of Chemistry, Shahid Bahonar University of Kerman, P.O. Box 76169-133, Kerman, Iran; 2 Young Researchers Society, Shahid Bahonar University of Kerman, P.O. Box 76175-133, Kerman, Iran

**Keywords:** Biological techniques, Biotechnology, Chemical biology, Plant sciences, Chemistry, Nanoscience and technology

## Abstract

*Bacillus thuringiensis* (*Bt*) were used for biosynthesis of amorphous TiO_2_ converted to distinct polymorphs (anatase, rutile, mix) under different temperature conditions. Characterizations of TiO_2_ nanoparticles were performed by using X-ray diffraction spectroscopy (XRD), Fourier-transform infrared spectroscopy (FTIR), scanning electron microscopy (SEM) and, energy-dispersive X-ray spectroscopy (EDX) analysis. Stability of five formulations under ultraviolet (UV) radiation with spore viability and mortality test on *Ephestia kuehniella Zeller* larvae were investigated. TiO_2_(mix) showed the highest viabilities of 79.76% after exposure to ultraviolet (UVA385 nm), while viabilities of non-protected spores under these conditions were 41.32%. The mortality of TiO_2_(mix), TiO_2_(anatase), TiO_2_(rutile), TiO_2_(amorphous) and free spore formulations on second-instar larvae of *Ephestia kuehniella* were 73.76%, 71.24%, 57.12%, 51.32%, and 50.32%, respectively on the 10th day of the experiment. The obtained results suggest that TiO_2_(amorphous) does not increase *Bt* resistance, but both phases of TiO_2_ nanoparticles synthesized (anatase and rutile) through the *Bacillus thuringiensis* and phase mixture can increase the persistence of *Bt* to the UV light. Furthermore, the combination of both crystalline phases of TiO_2_(mix) has the highest performance in improving the *Bt* resistance.

## Introduction

The most widely used microbial insecticides are those based on the bacterial pathogen *Bacillus thuringiensis* (*Bt*) used as a prosperous biological insecticide and an alternative to chemical pesticides for many years. It has been applied widely in pest management in forestry, agriculture and public health because of its safety to humans, animals, and the environment^1^. *Bt* not only is harmless to human and non-targeted insects, but also is an entomopathogen that produces insecticidal crystal proteins that are toxic for lepidopteran, dipteran, or coleopteran larvae^2^. A significant pest in the milling industry is the Mediterranean flour moth (*Ephestia kuehniella Zeller* (Lepidoptera: Pyralidae)) that is convenient, particularly due to available, thermal resistant, and easy to breed^3^. Entomopathogens lose their persistence when exposed to the sunlight in the field. Despite the fact that insecticides containing *Bt* can be very useful for insect control in a variety of situations, the short persistence of *Bt* agents after the application has become a primary influencing factor for its subsequent development^4^. Variable environmental factors, such as rain, UV radiation, and temperature, leads to microbial degradation or inactivation of the crystal proteins^2^. Most recently, several methods and studies are underway to develop different formulations for entomopathogens that can improve this activity and retain the microorganisms from solar degradation and other the harmful effects of the environment. Therefore, to overcome these limitations, such as susceptibility to light, short remnant lifetime and other conditions, optimization of *Bt* toxin formulation by nanoparticles is an essential parameter for its commercial production^5^. Ideally, the formulation should provide a maximum protective effect to the active agent, while it should have no antibacterial impact or the least antibacterial effect^6^.

Titanium dioxide (TiO_2_) has become a whole part of nanotechnology because of its many abilities to act as a photocatalyst, UV absorber and assist environmentally useful reactions. TiO_2_ occurs as three polymorphs, rutile, anatase, and brookite. However, because of the difficulty in its synthesis, brookite is less frequently introduced as photoactive material^7–10^. The bandgap of TiO_2_ corresponds to about 3.0 eV for rutile and around 3.2 eV for anatase and it can just absorb ultraviolet light (UV)^11^. Despite the more significant band gap of anatase compared with rutile, it is observed that the photoactivity performance of anatase generally is considered more than rutile. This is attributed to factors that improved performance including a higher density of localised states and consequent surface-adsorbed hydroxyl radicals and lower charge carrier recombination in anatase relative to rutile^12^.

On the other hand, the photoactivity of the pure phased is smaller than the phase mixture of different polymorphs of TiO_2_. However, for a long time, it is generally agreed that anatase exhibits a higher photoactivity compared to rutile TiO_2_. These polymorphs show several photoactivity performances due to their different properties^13–16^.

According to previous researches, amorphous TiO_2_ nanoparticles are not photoactive^17^. Rapid recombination of photogenerated electrons and holes before they can be involved in relevant reactions due to the existence of any defects in the amorphous phase leads to photochemical inactivity^17^. In this study, we develop a cost-effective and environmentally friendly approach method to use *Bt* for the biosynthesis of TiO_2_ nanoparticles. The effect of different polymorphs of TiO_2_ nanoparticles as a material to increase the persistence of *Bt* active agents against ultraviolet radiation was investigated.

## Results

### Structure and properties of TiO_2_

**Figure 1:**
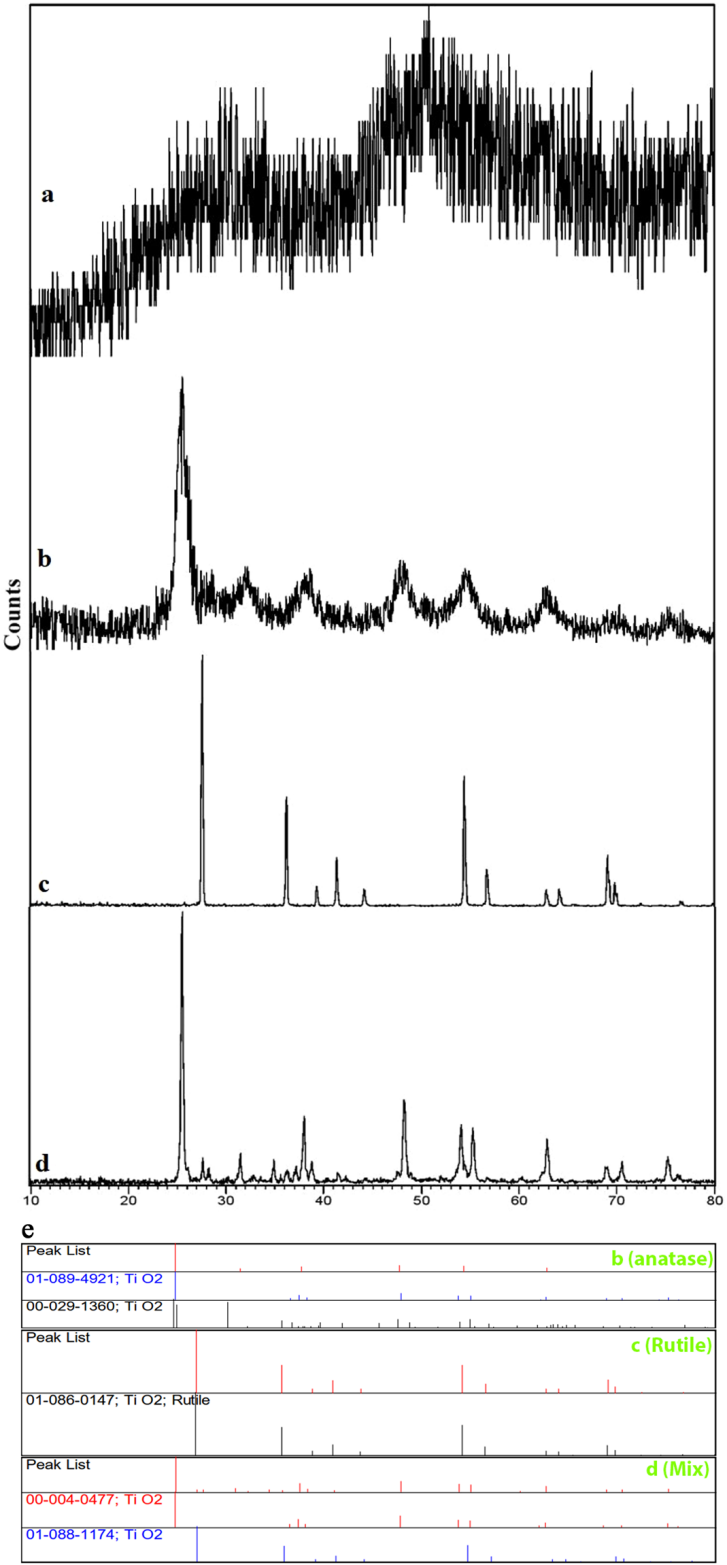
XRD pattern of (**a**) TiO_2_ (amorphous), (**b**) TiO_2_ (anatase), (**c**) TiO_2_ (rutile), (**d**) TiO_2_ (mix). (**e**) PDF card of (**b**) TiO_2_ (anatase), (**c**) TiO_2_ (rutile), (**d**) TiO_2_ (mix).

X-ray diffraction analysis was used for determining the crystalline phase of TiO_2_ nanoparticles. As seen in Fig. 1a, the XRD data of TiO_2_(amorphous) indicated any discernible reflection pattern. Figure 1b shows the XRD pattern of TiO_2_(anatase) powder (JCPDS: 01-073-1764). Broad peaks observed at values of 2*θ = *25.38, 32.00, 38.11,48.04, 54.56,62.93, 70.56 and,75.37° were equivalent to the planes (101), (004), (200), (105), (204), (116) and (215) and indicated the tetragonal structure of anatase TiO_2_ nanoparticles. This confirms the occurrence of a transition from amorphous to crystalline anatase phase at 450 °C. There is a significant correlation between the peaks from the TiO_2_ sample spectra and the library spectra of two different phases of TiO_2_. Figure 1c shows the XRD pattern of TiO_2_(rutile) powder (JCPDS:01-086-0147). The sharp peaks obtained at 2*θ* values equal to 27.53, 36.17, 39.30, 41.33, 44.15, 54.42, 56.73, 62.82, 64.14, 69.09 and 69.84° corresponds to the rutile structure. Figure 1d shows the XRD pattern of TiO_2_(mix) (JCPDS:00-004-0477) which includes a mix of peaks related to both phases. It is proved that the crystallinity increased with increasing calcination and, also the anatase phase emerges 450 °C, while conversion to rutile appears between 800 °C to 1200 °C^18^.

XRD patterns show that the peaks of TiO_2_(anatase) steadily become sharper and narrower with calcination temperature until T = 1200 °C, at which transition toTiO_2_(rutile) is completed. Although, the transformation from anatase to rutile starts typically at much lower temperatures, around 600 °C. As the XRD analysis confirms, a mixture of phases is observed at 800 °C (Fig. 1c).

Considering the correction in FWHM for the line broadening of the instruments, the average particle size (D) was computed using the Scherrer equation (Eq.1):

1 $$D=\frac{0.9{\rm{\lambda }}}{\beta cos\theta }$$

where *λ* is the X‐ray wavelength of radiation, *β* is FWHM in radians and *θ* is the Bragg’s angle. The crystallite size of the TiO_2_ (anatase) and TiO_2_ (rutile) is 15.7 and 79.24 nm, respectively. Figure 1e shows a PDF card of the crystalline phase of TiO_2_ nanoparticles.

**Figure 2:**
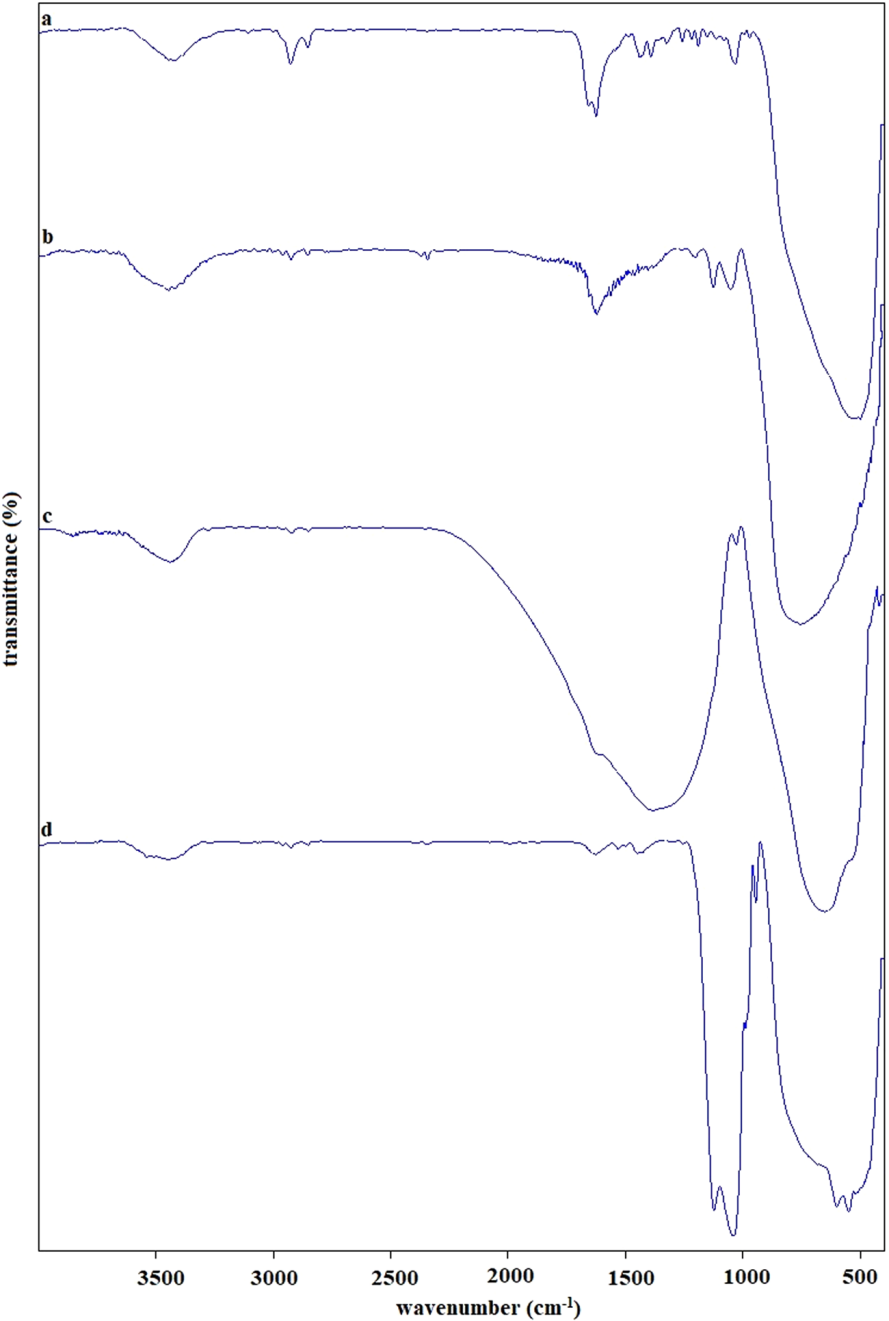
FT-IR spectra of (**a**) TiO_2_ (amorphous), (**b**) TiO_2_ (anatase), (**c**) TiO_2_ (rutile), (**d**) TiO_2_ (mix).

The FTIR spectra of *B*. *thuringiensis* synthesized TiO_2_ nanoparticles which according to XRD results in an amorphous structure, exhibited peaks at 3423, 2925, 1625, 1438, 1032, 500 cm^−1^ (Fig. 2a). A broad peak at 3430 cm^−1^ shows O-H stretching due to the phenolic and alcoholic group. The band at 2925 cm^−1^ in nanoparticles is corresponding to the symmetric stretch (C–H) of CH_3_ and CH_2_ groups of aliphatic chains. The peak at 1625 cm^−1^ indicates the presence of C=C ring stretching. The band observed at 1438 cm^–1^ is because of bending vibration of the CH_2_ in the lipids and proteins. The peak in the range of 1257–1020 cm^−1^ corresponds to the C-O stretching of alcoholic and carboxylic groups. The band at 1032 cm^−1^ assigned to the C-N stretching vibrations of aliphatic amines. In TiO_2_ (anatase) structure broadband observed in the range of 3600–3200 cm^−1^ is assigned to stretching hydroxyl group (O–H), representing the presence of surface water as moisture. The broad bands at 3445 and 1622 cm^−1^ observed in TiO_2_ (anatase) structure spectra are related to the Ti-OH stretching modes (Fig. 2b). In the TiO_2_ (rutile) structure the Ti–OH vibration band becomes much weaker and the full removal of the absorption peak at 1622 cm^−1^ infer the absence of hydroxyl groups in TiO_2_ (rutile) structure (Fig. 2c) and, as expected, in TiO_2_ (mix) structure, the peaks of both anatase and rutile were seen (Fig. 2d).

**Figure 3:**
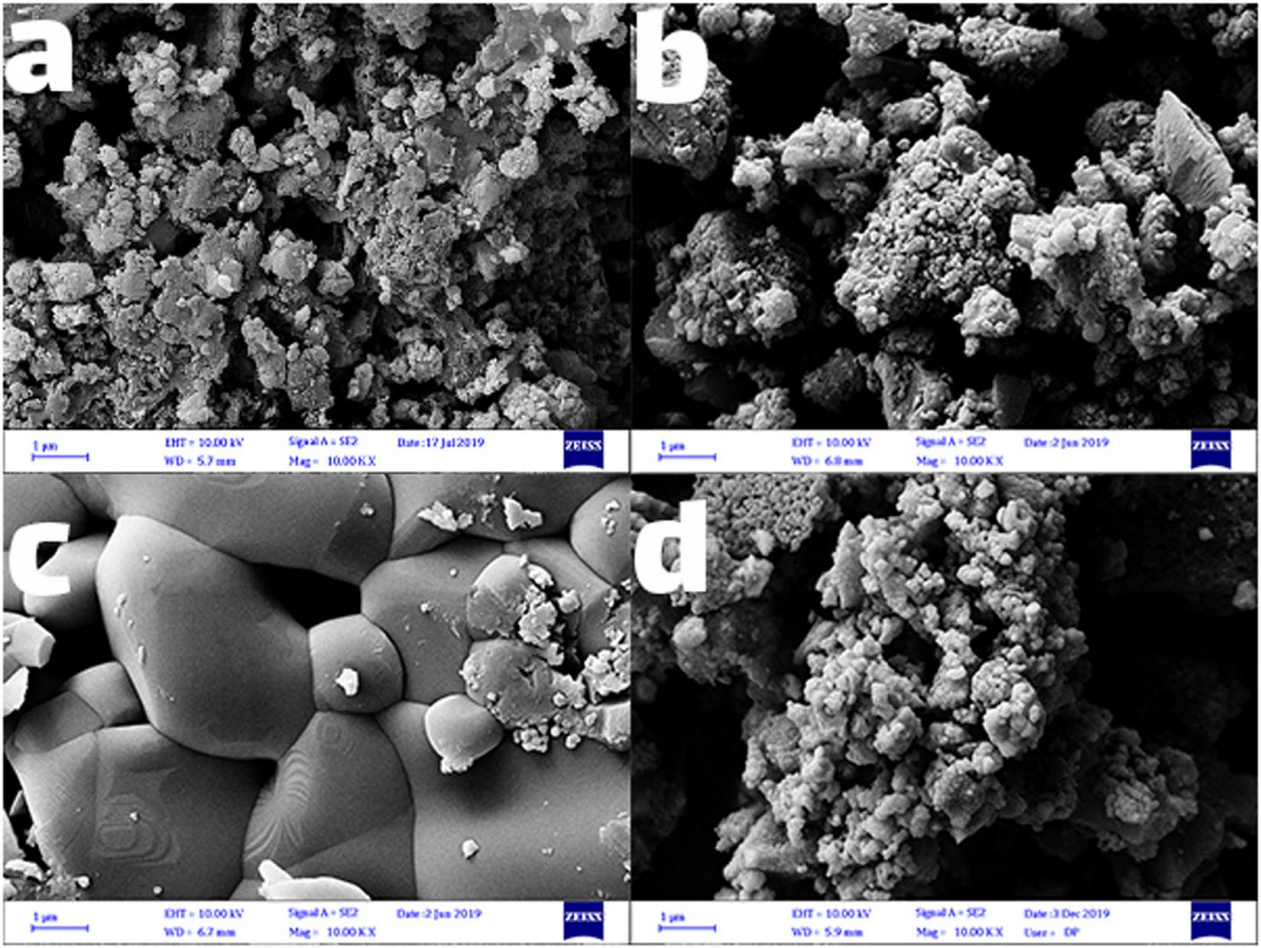
Field emission scanning electron microscopy (FE-SEM) images of (**a**) TiO_2_ (amorphous), (**b**) TiO_2_ (anatase), (**c**) TiO_2_ (rutile), (**d**) TiO_2_ (mix).

Figure 3 shows the morphology of the TiO_2_ nanoparticles at different temperatures. The SEM images of the synthesized TiO_2_ nanoparticles by *B*. *thuringiensis* show spherical clusters of the nanoparticles. Figure 3a is an image of the amorphous TiO_2_ powder. As seen, there is no definite morphology, and just disordered agglomerates with different sizes appeared. Figure 3b presents the images of TiO_2_ nanoparticles calcined at 450 °C, which demonstrate the small size of the particle. TiO_2_ nanoparticles were irregular spherical structure, oval in shape, spherical, and a few aggregates having a size of 33–44 nm. The width of the anatase peak diffraction from XRD confirms the smaller crystalline size at 450 °C. By increasing temperature to 1200 °C, the size increases, and the grain growth becomes remarkable (Fig. 3c), which describes the effects of heat treatment on the particle size of TiO_2_ nanoparticles. Figure 3d shows the images of TiO_2_ nanoparticles calcined at 800 °C. As you can see, it contains two different phases of TiO_2_ nanoparticles.

**Figure 4:**
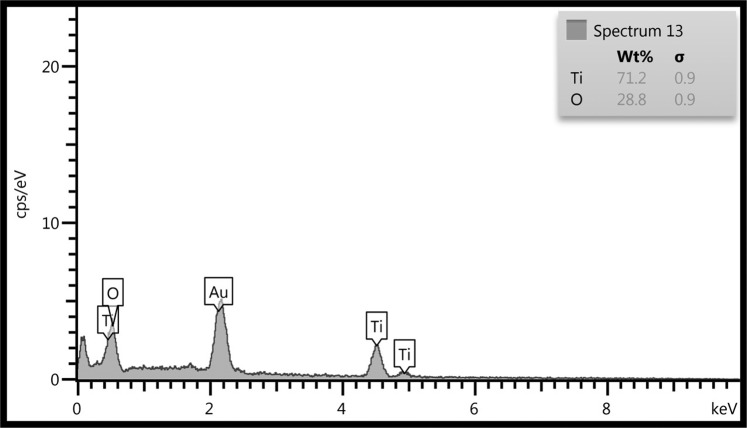
Energy-dispersive x-ray diffraction (EDX) spectrum of TiO_2_ (anatase and rutile).

Energy-dispersive X-ray spectroscopy (EDX) analysis of TiO_2_ nanoparticle (anatase and rutile) illustrates peaks for Ti element and oxygen. As shown in Fig. 4, there is no trace of any other impurities in the EDX analysis.

### Effect of UV radiations on spore viability

**Figure 5:**
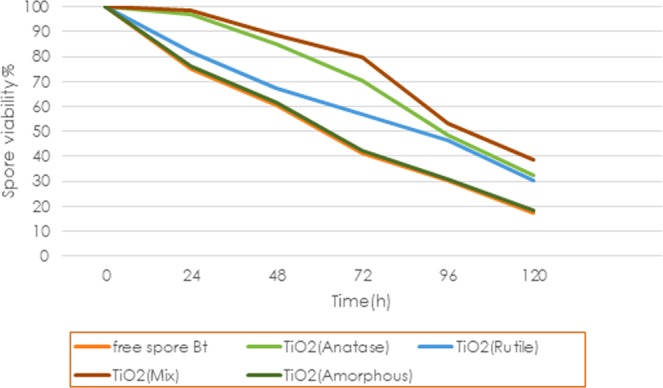
Effect of the time of UV-A irradiation on spore viability of *Bt* mixture of distinct polymorphs of TiO_2_ nanoparticles.

The effects of different polymorphs of TiO_2_ on the viability of *B*. *thuringiensis* formulations following exposure to UVA (385 nm) irradiation for five days are summarised in Fig. 5. After 72 h, the spore viability decreased from their initial values (100%) and reached to 79.76, 70.36, 56.63, 42.20, and 41.32% for TiO_2_ (mix), TiO_2_ (anatase), TiO_2_(rutile), TiO_2_(amorphous) and non-protected formulation, respectively. According to the results, there is a significant difference between the crystalline phase of TiO_2_ and the free spore of *Bt*, but the difference between TiO_2_(amorphous) and the free spore of *Bt* is not significant. TiO_2_(rutile) displays less protection of spore viability than TiO_2_(anatase) and TiO_2_(mix) sample shows the maximum protection of spore viability. Our results suggest that a mixture of anatase and rutile formulation could be a proper perspective for improving persistence and subsequently modifying the performance of *Bt* against UVA. This can be attributed to the photoactivity of each polymorph. The photoactivity of TiO_2_ is forcefully dependent on its crystallite size, phase structure, specific surface areas, and pore structure^19^. Photoactivity improved with increasing calcination temperature from 450 °C to 800 °C, which is when converting anatase to rutile. Larger grain size, lower specific surface areas and having a worse surface adsorption content of rutile causes lower photoactivity than anatase. As the results indicate, TiO_2_(rutile) formulation than less protection of spore viability than TiO_2_(anatase) formulation. Whereas anatase shows a higher photoactivity than rutile and amorphous phase with a higher level of adsorbed radicals that because of a more top surface area as well as a higher photoactivity per unit of surface area and as it was observed the protective effect of TiO_2_(anatase) formulation is greater than that of TiO_2_(rutile) and TiO_2_(amorphous) formulations.

A combination of two polymorphs of TiO_2_ improves the photoactivity because of the more effective spatial separation of the photoinduced electron-hole pair which been reported to display enhanced photoactivity relative to single-phase TiO_2_. It is worth mentioning that this is an outcome of better charge carrier separation, maybe through the trapping of electrons in TiO_2_(rutile) and the effective reduction in the rate of recombination of charge carrier^20,21^.

According to our previous research results, among all examined formulation, GO/olive oil formulation has the highest protection of spore viability^5^. The continuation of formulation’s exposure to UV radiations up to 72 h showed that spore viability for GO/olive oil formulation declined to 69.9%. While spore viability in two formulations of TiO2(mix) and TiO2(anatase) is more than the same parameter for GO/olive oil formulation. Thus, the sensitivity of Bt active agent to UV radiations limits its persistence in field conditions and justifies the addition of UV protectants in formulations for improving the stability and efficacy in the environment

### Mortality

**Figure 6:**
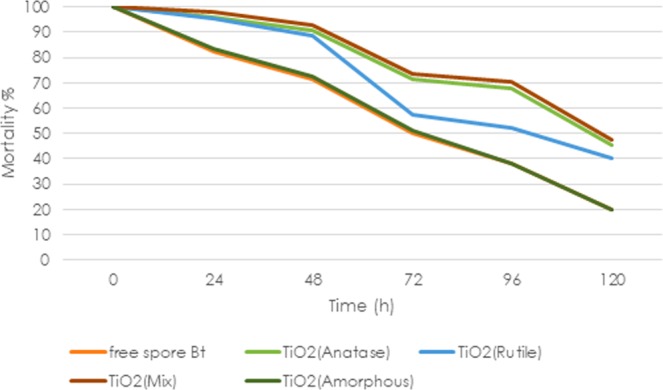
Effect of the time of UV-A irradiation on mortality of larvae of *Bt* mixture.

As seen in Table 1, the larval mortality of irradiated TiO_2_(mix), TiO_2_(anatase), TiO_2_(rutile), TiO_2_(amorphous) and non-protected formulation, after 72 h exposure to UVA radiation were 73.76%, 71.24%, 57.12%, 51.32%, and 50.32%, respectively (Fig. 6). The results proved that there was a significant difference between the four treatments (Duncan test, P < 0.05), whereas there was no significant difference between TiO_2_(amorphous) formulation with non-protect *Bt* formulation at p = 0.05. On the other hand that no protection was observed in the case of TiO_2_(amorphous) formulation. Among the four formulations, TiO_2_(mix) showed the maximum performance in mortality of larvae which is confirmed by difference in photoactivity of different polymorphs of TiO_2_. According to the results found by Poszgay *et al*., after 40 h of exposure to UV activity of *Bt* well is lost^22^. However, the spores and crystals of TiO_2_ formulation were able to remain active after 72 hours.

**Table 1:** The bioassay carried out on *Ephestiakuehniella*.

Treatment	Spore viability %	Mortality %
Non-irradiated no protected Bt	100 ± 0.00^a^	100 ± 0.00^a^
Irradiated non protected Bt	41.32 ± 0.96^e^	50.32 ± 0.96^e^
Non-irradiated TiO_2_(anatase) formulation	100 ± 0.00^a^	100 ± 0.00^a^
Irradiated TiO_2_(anatase) formulation	70.36 ± 0.85^c^	71.24 ± 1.36^c^
Non-irradiated TiO_2_(rutile) formulation	100 ± 0.00^a^	100 ± 0.00^a^
Irradiated TiO_2_(rutile) formulation	56.63 ± 1.64^d^	57.12 ± 0.63^d^
Non-irradiated TiO_2_(mix) formulation	100 ± 0.00^a^	100 ± 0.00^a^
Irradiated TiO_2_(mix) formulation	79.76 ± 1.84^b^	73.76 ± 0.76^b^
Non-irradiated TiO_2_(amorphous) formulation	100 ± 0.00^a^	100 ± 0.00^a^
Irradiated TiO_2_(amorphous) formulation	42.20 ± 0.83^e^	51.32 ± 0.42^e^

Note: Mean is the average of three replicates 45 larvae per in treatment, F = 1219.471, df = 10, p = 0.0001. Spore count for treatments was carried out using three replicates. F = 735.054, df = 9, p = 0.0001. Means within the same column, followed by a different letter are significant at p < 0.05, Duncan test. The data are mean ± SE.

## Discussion

*Bt* is applied to kill many different insects and has been widely for pest control, and a commercial formulation of this has been used as an alternative to chemicals. Sunlight as one of the most important natural environmental stress is known to inactivate the biopesticide preparations based on Bt because of UV radiation. According to library studies, we have applied a hitherto unreported, inexpensive, modern material, non-toxic for the quick synthesis of TiO_2_ nanoparticles. The protective effects of different polymorphs of TiO_2_ after exposure to UVA radiation were verified by the spore viability and crystal activity. Moderate protection has been shown by using TiO_2_(anatase) and TiO_2_(rutile), while TiO_2_(mix) formulation of *Bt* improved the resistance of the spores to UV irradiation. Therefore, synthesized TiO_2_(Mix) nanoparticles has the best performance in resisting *Bt* against UVA.

## Methods

### Materials

Titanium(IV) ethoxide (Ti(OC_2_H_5_)_4_) were purchased from Sigma-Aldrich and used without any purification prior to preparing TiO(OH)_2_. nutrient agar was purchased from Merck Chem. Co. (Germany). Deionized (DI) Water applied throughout all experiments and was purified with the Millipore system. *B*. *thuringiensis* subsp. Kurstaki KD-2 was obtained from Green Biotech (Tehran, Iran).

### Biosynthesis of TiO_2_ nanoparticles using *Bt*

TiO(OH)_2_ was used as a precursor for the biosynthesis of TiO_2_, where it was synthesized according to the previous references^23^. In summary, it was prepared by adding Ti(OC_2_H_5_)_4_ to water as a molar ratio of 1: 26.3. The mixture was stirred for 6 h at 25 °C. Afterwards, the resulting precipitate was filtered and washed several times with deionized water and, ethanol then dried in an oven at 90 °C overnight^24^.

For the biosynthesis of TiO_2_ nanoparticles, mother culture is provided after growing the *Bt* cells as a suspension culture in sterile distilled water containing suitable carbon and nitrogen source for 48 h, and this was treated as a mother culture. Then, 50 mL of this solution was diluted by adding 150 mL of sterile distilled water containing nutrients. Next, this solution was again allowed to grow for another 24 h. After adding 40 mL of 0.025 M TiO(OH)_2_ solution to the culture solution, it was heated for 30 min on steam bath up to 60 °C until white precipitation emerges at the bottom of the flask. The culture solution was incubated at 21 °C in the laboratory condition. After 48 h, the culture solution was observed to have separately white clusters deposited at the bottom of the flask^25^.

Further, the precipitate was filtered, washed with deionized water and, dried at 90 °C. After calcination, the amorphous phase of TiO_2_ was transformed into the crystalline phase. Different fractions of the obtained powder were calcinated at different temperatures 450 °C, 800 °C, and 1200 °C for 3 h, for the synthesis of TiO_2_(anatase), TiO_2_(mix), TiO_2_(rutile), respectively.

### Characterization of synthesized TiO_2_ nanoparticles

The X-ray spectra were recorded on a Phillips X’Pert PRO using filtered Cu Kα radiation (λ = 1.54178 Å) over the range 10° < 2*θ < *80°. Fourier transform infrared (FT-IR) spectra (4000–400 cm^−1^) by a Tensor 27 spectrometer (Bruker, Saarbrucken, Germany) were also used to confirm the TiO2 phase. SEM analysis of nanoparticles and nanocomposite were performed with a scanning electron microscope (FE-SEM, Sigma, Zeiss) equipped with energy dispersive X-Ray (EDX) elemental composition analyzer.

### Preparation of nano-formulation

After dispersing 0.01 g of different polymorphs of TiO_2_ in 100 mL sterile distilled water with ultrasonic vibration for 1 h, the mother culture of *Bt* cell (prepared in the previous section) was added and placed on a shaker set in a dark place for 24 h^5^.

### Evaluating of spores viability

Radiation was supplied by UVA tubes (Philips, 15 W, white light 385 nm peak emission) mounted 180 mm above the open Petri dishes. 40 mL of each formulation of different polymorphs of TiO_2_ were put on Petri dishes in triplicate. They were then exposed to UVA irradiation for 24, 48, 72, 96 and 120 h. Spore count of these samples was carried out by serial dilution with nutrient agar medium (CFU). Via serial dilution of formulations spread in Petri dishes containing nutrient agar spore counts was carried out and incubated at 28 °C for 24 h. The percentage of spore viability was estimated according to Eq. 2:

2 $${\rm{s}}=[{{\rm{N}}}_{{\rm{x}}}/{{\rm{N}}}_{0}]$$

where N_x_ is a number of the irradiated spores (free formulation or nano), and N_0_ is the number of the initial non-irradiated spores (free spores or nano)^2^.

### Bioassay

The toxicity of the four different formulations of TiO_2_ (irradiated and non-irradiated) and free spore (irradiated and non-irradiated) on second-instar larvae of *Ephestia kuehniella Zeller* was investigated. These larvae were reared on a diet containing a mixture of wheat bran, wheat flour under a long day (8 h dark, and 16 h light) at 28 °C with 60% relative humidity. After separating the second-instar larvae from flour medium, for the bioassay test six peanut pieces were soaked in 35 mL of the different formulations under sterile conditions for 4 min as larval food and then dried and placed in Petri dishes with 15 larvae. Next, the Petri dishes were incubated at 27 °C and 60% humidity. Mortality was recorded every 24 h and compared to the control for 10 days. Each treatment was carried out in triplicate^26^.

### Statistical analysis

All tests were performed using the Statistical Package for the Social Sciences software (SPSS 1998). All of the treatments were the average of three replicates of separate runs and compared using the Duncan test after analysis of variance (ANOVA).

## References

[1] Jozani GRS, Komakhin RA, Piruzian ES (2005). Comparative study of the expression of the native, modified, and hybrid cry3a genes of Bacillus thuringiensis in prokaryotic and eukaryotic cells. Russ. J. Genet..

[2] Jallouli W, Sellami S, Sellami M, Tounsi S (2014). Efficacy of olive mill wastewater for protecting Bacillus thuringiensis formulation from UV radiations. Acta Trop..

[3] Bouka H, Chemseddine M, Abbassi M, Brun J (2001). La pyrale des dattes dans la région de Tafilalet au Sud-Est du Maroc. Fruits.

[4] Jouzani GS, Valijanian E, Sharafi R (2017). *Bacillus thuringiensis*: a successful insecticide with new environmental features and tidings. Appl. Microbiol. Biotechnol..

[5] Maghsoudi S, Jalali E (2017). Noble UV protective agent for *Bacillus thuringiensis* based on a combination of graphene oxide and olive oil. Sci. Rep..

[6] Zhang, L. *et al*. A new formulation of *Bacillus thuringiensis*: UV protection and sustained release mosquito larvae studies. *Nat*. *Publ*. *Gr*. 1–8, 10.1038/srep39425 (2016). 10.1038/srep39425 PMC5177894 28004743

[7] Widaryanti, H. N. & Darminto. Fabrication of TiO_2_ nanoparticles and films and their UV-VIS absorbance. In *AIP Conference Proceedings*, vol. 13, 11–13 (2013).

[8] Reyes-Coronado D (2008). Phase-pure TiO_2_ nanoparticles: anatase, brookite and rutile. Nanotechnology.

[9] Di Paola A (2008). Photocatalytic activity of nanocrystalline TiO2 (brookite, rutile and brookite-based) powders prepared by thermohydrolysis of TiCl4 in aqueous chloride solutions. Colloids Surfaces A Physicochem. Eng. Asp..

[10] Yelamanchili, R. S. *New Approaches to the Synthesis of Porous and/or High Surface Area Transition Metal Oxides* (2008).

[11] Pantaroto Heloisa N., Ricomini-Filho Antonio P., Bertolini Martinna M., Dias da Silva José Humberto, Azevedo Neto Nilton F., Sukotjo Cortino, Rangel Elidiane C., Barão Valentim A.R. (2018). Antibacterial photocatalytic activity of different crystalline TiO2 phases in oral multispecies biofilm. Dental Materials.

[12] Luttrell, T. *et al*. Why is anatase a better photocatalyst than rutile? - Model studies on epitaxial TiO2 films. *Sci*. *Rep*. 1–8, 10.1038/srep04043 (2014). 10.1038/srep04043 PMC3918909 24509651

[13] Hanaor Dorian A. H., Sorrell Charles C. (2010). Review of the anatase to rutile phase transformation. Journal of Materials Science.

[14] Kawahara T (2002). A patterned TiO_2_ (anatase)/TiO_2_ (rutile) bilayer‐type photocatalyst: effect of the anatase/rutile junction on the photocatalytic activity. Angew. Chemie Int. Ed..

[15] Di Paola A, Bellardita M, Ceccato R, Palmisano L, Parrino F (2009). Highly active photocatalytic TiO_2_ powders obtained by thermohydrolysis of TiCl4 in water. J. Phys. Chem. C.

[16] Cappelletti G, Bianchi CL, Ardizzone S (2008). Nano-titania assisted photoreduction of Cr (VI): the role of the different TiO_2_ polymorphs. Appl. Catal. B Environ..

[17] Krylova G, Na C (2015). Photoinduced crystallization and activation of amorphous titanium dioxide. J. Phys. Chem. C.

[18] Hun OS, Seong Kim J, Suk Chung J, Jung Kim E, Hong Hahn S (2005). Crystallization and Photoactivity of TiO_2_ Films Formed on Soda Lime Glass by a Sol-Gel Dip-Coating Process. Chem. Eng. Commun..

[19] Atta Supriya, Pennington Ashley M., Celik Fuat E., Fabris Laura (2018). TiO2 on Gold Nanostars Enhances Photocatalytic Water Reduction in the Near-Infrared Regime. Chem.

[20] Wang, G., Xu, L., Zhang, J., Yin, T. & Han, D. Enhanced photocatalytic activity of powders (P25) via calcination treatment. *Int*. *J*. *Photoenergy* **2012** (2012).

[21] Scanlon DO (2013). Band alignment of rutile and anatase TiO_2_. Nat. Mater..

[22] Saxena D (2002). A UV tolerant mutant of Bacillus thuringiensis subsp. kurstaki producing melanin. Curr. Microbiol..

[23] Tuwati A, Fan M, Russell AG, Wang J, Dacosta HFM (2013). New CO_2_ sorbent synthesized with nanoporous TiO (OH)_2_and K2CO3. Energy & Fuels.

[24] Irani M, Gasem KAM, Dutcher B, Fan M (2016). CO_2_ capture using nanoporous TiO (OH)_2_/tetraethylenepentamine. Fuel.

[25] Kirthi AV (2011). Biosynthesis of titanium dioxide nanoparticles using bacterium Bacillus subtilis. Mater. Lett..

[26] Khorramvatan S, Marzban R, Ardjmand M, Safekordi A, Askary H (2014). The effect of polymers on the stability of microencapsulated formulations of *Bacillus thuringiensis* subsp. kurstaki (Bt-KD2) after exposure to Ultra Violet Radiation. Biocontrol Sci. Technol..

